# Hot of Not: Physiological versus Meteorological Heatwaves—Support for a Mean Temperature Threshold

**DOI:** 10.3390/ijerph13080753

**Published:** 2016-07-26

**Authors:** Matt Luther, Fergus W. Gardiner, Claire Hansen, David Caldicott

**Affiliations:** 1 Calvary Hospital Bruce Cnr Belconnen, Way & Haydon Drive, Bruce, ACT 2617, Australia; matt.luther@calvary-act.com.au (M.L.); claire.hansen@calvary-act.com.au (C.H.); david.caldicott@calvary-act.com.au (D.C.); 2 Australian Catholic University, 223 Antill St, Watson, ACT 2602, Australia; 3 University of Canberra, University Dr, Bruce, ACT 2617, Australia; 4 School of Biomedical Sciences, Charles Sturt University, Wagga, NSW 2650, Australia; 5 ANU Medical School, Australian National University, Building 4, The Canberra Hospital, Hospital Rd, Garran, ACT 2605, Australia

**Keywords:** heat, heatwave, pathophysiological, meteorological, alert tool, plan, alert fatigue, climate change, extreme weather

## Abstract

The aim of this study was to determine whether a revised heat warning threshold provides an enhanced predictive tool for increases in Emergency Department heat-related presentations in Canberra, Australia. All Emergency Department triage records containing the word “heat”, as well as those diagnosing a heat related illness for the summer periods 2013/2014, 2014/2015, and 2015/2016 were searched. Then a medical record review was conducted to confirm that the patient’s presentation was related to environmental heat, which was defined by the final clinical diagnosis, presentation complaint and details of the patient’s treatment. Researchers then compared this presentation data, to a mean threshold formula. The mean threshold formula included the past three consecutive daily mean temperatures and the last measured temperature upon presentation. This formula was designed to take into account the variance of night-time lows, with concurrent daily ambient temperatures, and was used to determine whether there was a correlation between heat-related presentations and increasing mean temperatures. Heat-related presentations appeared to occur when the mean threshold temperature reached 25 °C (77 °F), with significant increases when the mean threshold reached 30 °C (86 °F). These results confirm that a mean temperature of 30 °C corresponds to a relevant local public health heat-related threat.

## 1. Introduction

There has been significant research worldwide pertaining to the effects of heatwaves on local populations and individual countries’ associated warning systems, particularly post critical events, such as the Chicago and European heatwaves of 1995 and 2003 [1,2]. The Australian literature covers topics including planning, prediction of events, alert/awareness systems, population awareness of risk, stakeholder engagement, Emergency Department impact, spatial analysis and morbidity and mortality [1,3,4,5,6,7,8,9,10,11,12,13,14,15]. These topics generally follow the standard emergency management cycle: Preparedness, Response, Recovery, and Mitigation [16]. The existing literature appears limited, focusing mainly on large city populations with a coastal influence. There has been minimal literature addressing landlocked populations [8].

It is widely accepted that the definition of a heatwave is unique to meteorological influences such as regional seasonal average temperatures (maximum and minimum), and humidity, as well as population influences such as economic, social, cultural and health status [17,18]. A heatwave definition and the subsequent trigger thresholds associated with a public health heatwave human impact management plan cannot be arbitrary, and needs to be tailored and validated against local variations. Single point alert tools/triggers, or thresholds have difficulty in accounting for population variances and fluctuations in their associated health impact, related to temperature ranges throughout a 24-h period, and are generally unable to differentiate between physiological and meteorological events. On this basis, different strategies should be recommended for different population groups in differing geographical regions. For example, those at the extremes of the age, the very young or those with co-morbidities require different approaches. Mean daily temperatures should be used as threshold markers rather than isolated maximum temperatures in public health [1,8,11,12,13,15].

There is a need to differentiate between an extreme heat event and a heatwave within public health plans and subsequent alerts, considering the physiological variables involved in exposure to heat over time versus a single extreme exposure. A heatwave is typically defined as a period of excessively hot weather, whereas an extreme heat event usually arises from a single spike in temperature. Sustained excess bodily heat is unusually absorbed from high daytime temperatures that is not sufficiently dissipated overnight, due to high night-time temperatures. High night-time temperatures also lead to increased fatigue and frustration secondary to interrupted sleep patterns [18]. Heat stress arises from the inability of the human body to acclimatise to a higher base environmental temperature. Acclimatisation involves physiological adjustments of the cardiovascular, endocrine, and renal systems which can take up to 30 days to occur [18]. A heatwave should be considered as a period of at least three days where the combined effects of excess heat leads to heat stress; both maximum and minimum temperatures are used in this assessment of excessively hot weather [18].

Canberra, in the Australian Capital Territory, has an Extreme Heat Management Plan, that is triggered by a 3 day forecast of consecutive days with a maximum temperature of 35 °C (95 °F) or greater [19,20]. During January and February 2014, the Australian Capital Territory (ACT) Extreme Heat Management Plan was implemented three times over the periods; 14 to 18 January, 29 January to 4 February and 7 February to 10 February. During the subsequent summer periods; December 2014, January and February 2015, December 2015, January and February 2016, the ACT Extreme Heat Management Plan was not implemented. During the three activations of the ACT Extreme Heat Management Plan there were six heat-related cases noted (not requiring Emergency Department or hospital treatment), with no reported impact to business continuity at either of the two public Emergency Departments (there are only two Emergency Departments in the ACT with no private Emergency Department options).

The activation of the ACT Extreme Heat Management Plan cascaded to the subsequent implementation of the local individual health care provider’s Health Emergency Plans-Appendix [19,20]. The aim of this health plan/appendix is to assist the broader health sector in ensuring preparedness through a coordinated response to minimise/mitigate the associated risks while managing the consequence of such heatwave events [20]. According to the local plan, public hospitals within the ACT reference their own organisational plans, provide daily reporting of capability, supply status and enhanced monitoring of the events impact (in the form of a situational report to the local Health Emergency Management Unit). This information then enables the health department to ensure a coordinated territory wide response to the public health threat. Whilst this element of the plan operated efficiently and effectively during recent activations, the threshold point for initiation of the plan focuses on a single meteorological trigger which, when analysed against local patient presentation data, may not represent a validated threshold/trigger alert tool. During these activation periods there was a low-to-zero morbidity and zero mortality rate.

Heatwaves in France during August of 2003 led to 15,000 deaths, making it one of the greatest health catastrophes in France’s history [21]. A number of Australian cities have also experienced significant heat-related deaths (Table 1), though on a significantly smaller scale. This data indicates that current thresholds may not adequately reflect the degree of heat-related mortality within Australia. Changing the health heat warning system is important, as there appears to be a performance gap in the current system and the reported mortality associated with heat stress/stroke.

Many health warning systems do not adequately account for mean daily temperatures and do not adequately reflect how heatwaves affect the community. Mean threshold triggers have been identified as being better suited to adequately predict increased heat related presentations [1,13,15]. Nicholls et al. proposed an alert tool based on a daily mean temperature threshold of 30 °C (86 °F) [1]. This tool aids in the distinction between meteorological and physiological heatwaves through the recognition of the human body’s tolerance for environmental heat, tempered by lower overnight temperatures. Variables such as economic, social, cultural and health status cannot be captured in such a simple heat alert tool, though may be accounted for in a validated deviation in the mean temperature threshold for a specific population cohort. For example, if the population is generally healthy, has easy access to air-conditioned environments and is normally exposed to sustained higher local temperatures, then a higher daily mean threshold than 30 °C (86 °F) may be indicated.

The primary intent of this research is to broaden the literature associated with changing the current warning system, based on a threshold of three consecutive days of a temperature >35 °C (95 °F), to a mean 3-day temperature threshold of 30 °C (86 °F), taking into consideration night-time lows. The reasoning for this argued change in threshold, is associated with better preparing the Emergency Department to expect an increase in heat related presentations. The activation of the current system, of three consecutive maximum day temperatures above >35 °C (95 °F), have not resulted in any Emergency Department presentations related to heat. A mean 3-day temperature threshold, would allow better allocation of resources, increasing effectiveness while minimising the impact of heat related events. As such, the researchers aimed to test the following hypothesis: does a mean temperature of 30 °C (86 °F) correspond to an increase in heat related presentations, in the context of a socioeconomically stable inland population.

## 2. Methodology

### 2.1. Study Design and Context

A retrospective chart review was performed at a metropolitan Emergency Department seeing an average of 56,000 patients a year (2013/2014, 2014/2015, 2015/2016), in Canberra, Australia. At the last census (June 2013), the population of the Australian Capital Territory stood at 383,400 [22]. This study was approved by the Hospital Research Ethics Committee, approval code: 09-2016.

### 2.2. Participants and Data Collection

The local Emergency Department Information System (EDIS) was used to retrieve all Emergency Department triage information that contained the word “heat”, as well as those diagnosed with a heat related illness, for the summer periods (December, January, February) of 2013/2014, 2014/2015, and 2015/16. In reviewing the EDIS data, researchers recorded:

- The patient’s medical record number
- Patient’s home demographics (suburb or town)
- Arrival time and date

The last measured environmental temperature and the past 3-day mean temperature related to the presentation date, and was gathered from meteorological data for the period. This data was gained from the nearest weather observation station to the hospital: Queanbeyan Bowling Club (site number 070072); latitude 35.36° S, longitude 149.23° E. This was due to the closure of the Canberra Airport observation station in 2010.

Researchers determined whether the patient’s presentation was related to heat through analysis of the final Emergency Department clinical diagnosis, the presentation complaint and the patient’s treatment details. Valid heat related presentations included those highlighted in Table 2 (binary yes/no response), with all other presentations discounted from the study. To confirm whether the presentation was related to heat, against the digital record, researchers audited the patient’s notes, gained via the hospital’s medical records department, to determine the following:

- Did the presentation have signs and symptoms related and conducive to heat, and
- Did the signs and symptoms result in treatment for a heat related illness, and
- Was the final diagnosis upon discharge related to heat

The researchers recorded whether the patient presenting with a heat related complaint was within a high risk population (the very young (<2 years old), the elderly (>64 years old) and those with significant comorbidities). Allocation was determined if the patient adhered to the criteria highlighted in Table 3 and Table 4. Patients only needed to adhere to one of these characteristics, to be considered a high risk patient.

Researchers used a mean threshold formula. This formula takes into account the variance in night time lows with concurrent ambient temperatures. The current measure was believed to reflect the premise:

- That as temperature means progressively build over three days (heatwaves), patients are less likely to be able to cope, leading to increased hospital presentations [1,15,18], and
- Reflect studies [13,23,24] from major Australian cities that reported an increased mortality or morbidity, in relation to high ambient temperatures (heat events)

As such, researchers used the following formula:

(TDA + LMT)/2

where TDA = Last three day temperature average; LMT = Last measured temperature (09:00 or 15:00) before patient presentation.

This formula was modified from Nicholls et al. [1] to include a 3 day averaged mean temperature, as recommended by Nairn and Fawcett (2015) [18,25]. This formula was then adapted to account for the last patient presentation temperature. This was believed to take into consideration current ambient and consecutive temperatures [25]. The formula was then used to determine whether there was a correlation between patient heat presentations and increasing mean temperatures.

## 3. Results

**Figure 1:**
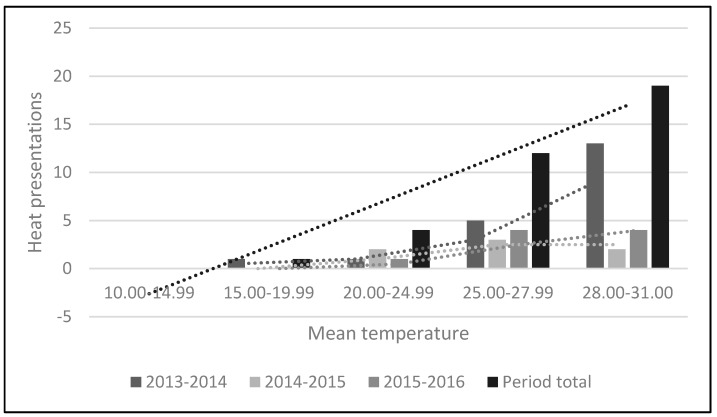
Mean temperature threshold graphical representation. The average linear trend lines increase the closer the mean temperature is to reaching 30.0 °C, and was significant (*p* = 0.01).

The results indicate that the last measured meteorological temperature before patient presentation to the Emergency Department averaged 32 °C (89.6 °F). Heat related presentations appeared to increase when the mean threshold temperature reached 25 °C (77 °F), with significant increases when the mean threshold reached 30 °C (86 °F) (Table 5; Figure 1). A Chi-square test of independence was performed (based on results highlighted in Table 5) to compare the mean threshold temperature formula (TDA + LMT)/2), to that of an expected 3 day temperature threshold above 35 °C. These results were statistically significant (*p* = 0.01), and confirm the hypothesis that a mean temperature of 30 °C (86 °F) corresponds to an increase in heat related presentations, representative of a potential public health emergency thus an indicative threshold.

The Australian Bureau of Meteorology recorded meteorological heatwave events (*n* = 3) during the local summer months of December 2013, January and February 2014; December 2014, January and February 2015; and of December 2015, January and February 2016. Yearly temperatures are highlighted in Table 6.

Seventy-eight (*n* = 78) patient records were identified as containing the word ‘heat’ within the presenting complaint or diagnosis, as a result of 168,000 Emergency Department triage presentations being assessed electronically. Subsequently, 42 participants were excluded (Table 7) from the study due to not being a valid heat related presentation (Table 2). Expansion of the health services impact concept, to a mean temperature of 30 °C (86 °F) found that at one of the territories emergency departments there were 36 patients with a heat related diagnosis (Table 8).

All 36 patients with a heat related presentation, were in the high risk category (Table 3 and Table 4). The total number of patients with a single risk factor equalled 23, with the remaining 10 patients having one or more heat risk factors. Multiple risk factors most commonly related to age, conducting strenuous outdoor activity and having limited access to air-conditioning (Table 9). The top 3 discharge diagnosis included, dehydration (*n* = 10), general heat related illnesses (*n* = 9) and heat stress (*n* = 7). The remaining diagnosis (*n* = 10) being a combination, detailed in Table 8. The primary symptom of a heat related illness was patient syncope (*n* = 17), with nausea and/or vomiting (*n* = 7), at 47.2% and 19.4% respectively (Table 10).

**Figure 2:**
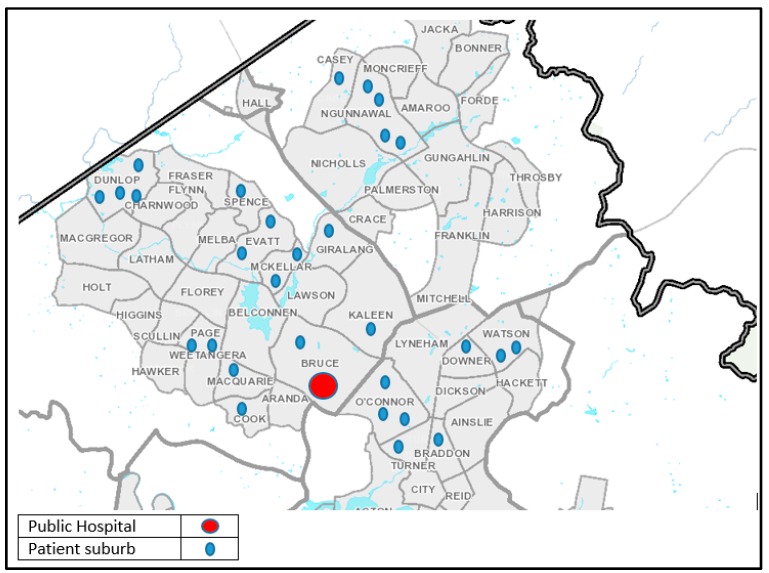
Patient residence results. This graph indicates that most of the patients resided in the northern aspects of the ACT. This is consistent with many patients presenting to the Canberra Hospital, located in south Canberra.

Results indicated that patient sociodemographic information varied. Patient age ranged from a 6 month old female to a 91 year old male. The mean age was 40 years (a female mean age of 36 years, and a male mean age of 43 years). The majority of patients resided in the ACT (*n* = 31), with a minority from interstate (*n* = 5). Most of the ACT residents were from the northern aspects of the ACT (*n* = 29), with the remaining (*n* = 2) from southern ACT (Figure 2). Of note, southern ACT has a large public hospital (The Canberra Hospital and Health Services), which provides healthcare for south residences, most likely explaining the predominance of northern aspect presentation.

There were no periods, during the observed period, where the mean daily temperature met or exceeded 30 °C (86 °F). Whilst there were multiple meteorological events where the predicted/actual maximum daily temperatures reach or exceeded 35 °C (95 °F), these were tempered by significantly lower overnight temperatures <22 °C (71.68 °F).

## 4. Discussion

Mean daily temperatures seem to provide a predictive tool. Table 5 and Figure 1 illustrate that mean daily temperatures exceeding 25 °C (77 °F) led to an increased likelihood of heat related presentations to the local Emergency Department, with a significant likelihood of presentations the closer the mean temperature was to reaching or exceeding 30 °C (86 °F). A mean temperature of 30 °C (86 °F) would thus appear to provide a useful threshold for issuing heat alerts in Canberra. This is consistent with previous research [1] and corresponds to alternate recommendations setting a Canberra threshold of 25.9 °C (78.62 °F) [18]. The results described could validate a simple method for public heat alert systems elsewhere, to be implemented regionally or within individual hospitals/healthcare facilities. The Australian Bureau of Meteorology provides online access to forecast weather trends, thus allowing public health clinicians the ability to determine the past 3-day average.

This is in contrast to a study by Barnett, Tony, and Clements (2010) [27] indicating that different temperature indicators, including mean, minimum, and maximum, have the same predictive ability in heat related mortality. Specifically, the current study found no heat-related presentations associated with a consecutive max temperature >35 °C (95 °F), whereas a mean threshold was significantly associated with increased heat-related presentations. Barnett, Tony, and Clements (2010) [27] found large differences in the best measurement measure between age groups, seasons, and cities, and that there was no one temperature measure that was superior to others overall (nationally). They recommended that new studies should chose temperature measures, based on practical concerns, as related to the area and data availability [27]. This current study, adheres to this recommendation, by applying a threshold measure that relates to the Canberra climate and patient demographic.

Changing Canberra’s activation process to a mean threshold, is reflective of other heat warning systems within other Australian states. This includes the 2015 Victorian Heat Health Plan, which details that a heat health warning should be communicated when the minimum and maximum average temperature is >30 °C (86 °F). This plan changed from using maximum thresholds, to basing its activation process on the Bureau of Meteorology’s *Heatwave Service for Australia* definition of heatwave as “three days or more of high maximum and minimum temperature that are unusual for that location” [28]. The South Australian Government has also moved away from an activation process of three consecutive days >35 °C (95 °F), and have specifically redefined their activation trigger to three or more consecutive days with an average daily temperature of >32 °C (90 °F). This figure was designed to consider cooler coastal winds. These changes provide further validation of a need to change the Canberra regions activation threshold to a mean temperature measure [29]. Furthermore, this is reflective of preceding research conducted in South Australia. One study found mortality was associated with heat events of three or more consecutive days with maximum temperatures >43 °C (109 °F), or an average temperature >34 °C (93 °F) [30], whereas another study [15] found that Emergency Department presentations became apparent above maximum and minimum temperatures of 34 °C (93 °F) and 22 °C (72 °F), with a mean presentation temperature of 28 °C (82 °F). These government changes and preceding studies, provide complying literature associated with the movement away from maximum temperature thresholds within Australia.

During the Canberra heatwave events of January and February 2014, the impact on the normal business of the Emergency Departments was distinguished as minimal, though it is noted that this view only highlights the requirement for a differentiation between physiological and meteorological heatwaves with such plans. As discussed, there are other indicators and variables associated with population resilience to heat based events such as the exacerbation of chronic conditions, an important consideration when creating/evaluating an extreme heat event plan or allocating a heat based threshold.

There is a risk associated with the setting of a “safe” (lower threshold) heat plan activation, in the form of alert fatigue both within the community and the health sector. The threshold must be set in an effort to minimise the public health risk presented to a community secondary to an extreme heat event, though due to the geographical breath and location of Australia, Australians are both accustomed to and aware of ‘hot summers’ and bushfire threats. Thus when considering public health heatwave/extreme heat notifications, if the threshold is too low, excluding those unaccustomed (visitors/tourists), there is a risk of alert fatigue where the recipients of the message experience a negative response (poor uptake of the message), when the message is delivered too frequently [31]. Further examination of heatwave events, including the experience of local medical officers/general practitioners and volunteer health care provider organisations, may help in eliciting a further understanding of this potential negative communication risk.

Complicating the physiological heatwave situation in Australian is the concurrent heightened bushfire risk that is also associated with sustained high meteorological temperatures. Not only do active bushfires have a direct impact on local meteorological conditions, they also complicate the public health situation with reduced air quality. These considerations should be taken into account, with other factors such as local population influences; economic, social, cultural and health status, in planning to minimise the impact of heat events [32].

Further factors such as wind speed and humidity play a defined role in physiological heatwaves events. The variance between coastal and inland threshold values/tools suggests such an influence, though further analysis is required to describe the variable. A more complex tool may be required to take these meteorological variables into account. It has been suggested that such variables may be addressed in a mean threshold range trigger, for example 27–30 °C (80.6–86 °F), with independent co-triggers around humidity and peak temperatures [18]. Emerging meteorological variables such as those noted secondary to climate change will also necessitate further analysis of heatwave threshold based plans.

**Figure 3:**
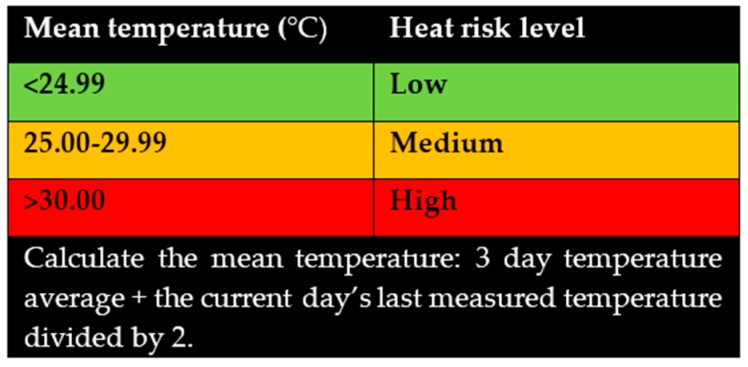
Community health heat risk table (Canberra, Australia). This table could be used as a community health warning system. This table would be in keeping with other heat warning systems, such as bush fire risk, presented on local news.

Whilst a portion of the literature suggests that general/widespread adoption of a mean daily temperature threshold is not likely in regions such as Europe, due to their local experience around the public health impact of heatwaves and extreme high temperatures events, this should not discourage the application of a mean temperature type alert system within Australia. Such a mean temperature threshold system takes into account both heatwaves and extreme temperature events when the mean threshold is set at a point appropriate to the regional seasonal average temperatures [33]. A notification tool, as outlined in Figure 3, could be developed and used in the notification of heat related health risks (Figure 3). This tool could be displayed in prominent areas within the community and on local television. Modern media modalities such as social media should also be considered with all public health messaging, capturing large sectors of a populations, within a very short period.

When deciding on or setting a trigger threshold for public health related heat events, health departments and agencies would be well advised to differentiate between physiological and meteorological heatwaves, as well as isolated extreme heat events. Such decisions need to take into account; the resilience of the local population, regional seasonal average temperatures, economic, social, cultural, and health status factors.

Within the defined local population/cohort, during the examined summer periods of sustained heat, there were significant outdoor mass gatherings, including the National Multicultural Festival and Fringe Festival. These events being held in Canberra on an annual basis. The outdoor nature and large participation in these public events presents the potential for a public health challenge/threat. Had there been an associated consequence, with significant heat related presentations, this may have presented a further example of physiological versus meteorological heatwaves. Fortunately, there were no significant heatwave related presentations to the local Emergency Departments, thus no there was no health impact noted in association with these large outdoor events.

A limitation of this study, could include that patients with low-moderate heat illnesses may not have been admitted or reported to the hospital. This could have therefore, resulted in an underreporting of the wider patient data, as this study only included those who presented to our Emergency Department. Further investigation into the health impact noted by general practitioners, prehospital healthcare providers and volunteer first aid providers, during a heatwave/extreme heat event, would expand on the complete public health picture. Furthermore this study does not directly address the aggravation of diseases related to heatwaves, or the possible deaths as a potential result of changes in temperature. For example, a future area of research could confirm the belief, that as the temperature climbs to >28 °C (82.4 °F), the risk of cardiac arrest increases. Future studies could also test the heat aggravations associated with chronic disease, such as acute coronary syndrome, renal disease, hypertension, and diabetes mellitus. It is believe that these sub-groups, and the associated commodities, are at greater risk for the ill effects from the heat.

## 5. Conclusions

Whilst there are many variables associated in setting a heatwave plan activation threshold, the minimal impact noted within this study, under the current Extreme Heat Management Plan, supports the application of a mean daily temperature based threshold, taking into account local factors such as socioeconomic variables. The population resilience experienced during the heatwaves of January and February 2014 is suggestive that the current plan trigger threshold of maximum daily temperatures reaching or exceeding 35 °C (95 °F) (actual or predicted) over three consecutive days, could be revised, considering the demonstrated tolerance. Results gained from this study validate existing mean threshold recommendations [1,15,18]. Further study into the influence of heatwaves and extreme heat events, outside of the established acute health care sector is indicated to further account for resilience and mass gathering impact.

## Figures and Tables

**Table 1:** 2011 heat-related deaths and estimated deaths per annum. Data indicates that as the population increases, so will the heat-related deaths. This could place healthcare under increased pressure. Source: PwC Australia [21].

City	2011	2030	2050
Brisbane	19	32	49
Perth	10	17	25
Sydney	18	25	34
ACT (Canberra)	2	4	6
Adelaide	11	16	21
Melbourne	23	33	48
Tasmania	3	4	5
Total	86	131	188

**Table 2:** Valid heat related presentations. Details mild and severe heat related illness, and the presenting symptoms, signs, and treatment.

Type	Symptoms	Signs	Emergency Treatment
Mild illness (cramps, exhaustion)	Diarrhoea, dizziness, headache, irritability, loss of coordination, nausea/vomiting, syncope, weakness	Core temperature <40 °C, normal mentation, goose flesh, pallor, tachycardia, hypotension	Oral rehydration Symptom management Monitoring
Heat stroke	Confusion, dizziness, hallucination, headache, nausea/vomiting, syncope	Core temperature >40 °C, altered mental status, hot skin with or without perspiration, hypotension, seizure, tachycardia	Intravenous rehydration Electrolyte monitoring Active cooling Seizure management Management/prophylaxis Monitoring

**Table 3:** Physiological and environmental risk factors for patients presenting with a heat-related illness. Details the demographic most at risk of heat-related emergency care.

Physiological and Environmental
Age older then >64 year old or younger then <2 year old
Cognitive impairment
Heart and lung disease
Limited access to air-conditioning
Mental illness
Obesity
Physical disability/impaired mobility
Poor fitness level
Sickle cell trait
Strenuous outdoor physical activity during hottest day-time hours
Urban built-up residence or living on higher floors

**Table 4:** Medication and recreation stimulant high risk factors for patients presenting with a heat-related illness. Medications and substances, such as these can increase heat susceptibility through dehydration and autoregulation.

Medication and/or Substances	
Alcohol	Laxatives
Alpha-adrenergic agonist	Neuroleptics
Amphetamines	Phenothiazine
Anticholinergics	Other stimulants
Antihistamines	Thyroid receptor agonists
Benzodiazepines	Tricyclic antidepressants
Beta Blockers	Diuretics
Calcium channel blockers	Ephedra-containing supplements
Cocaine	

**Table 5:** Research formula threshold findings. Heat-related presentations increased the closer the mean temperature was to reaching 30 °C (86 °F).

Heat Related Presentations				
Mean Temperature (°C)	2013–2014	2014–2015	2015–2016	Period Total
10.0–14.9	0	0	0	0
15.0–19.9	1	0	0	1
20.0–24.9	1	2	1	4
25.0–27.9	5	3	4	12
28.0–31.0	13	2	4	19
Total	20	7	9	36
Summer mean temperature (°C)	21.1	20.48	20.95	

**Table 6:** Temperatures (°C) throughout the study period. Source: Bureau of Meteorology [17,26].

Year	Summer Month	Mean Monthly Temperature	Max Temperature	Min Temperature	Number of Days Reaching 35 °C or Greater
2013	December	20.0	38.1	2.7	3
2014	January	21.8	40.2	6.1	9
	February	21.5	39.3	5.1	7
	December	20.2	32.7	7.2	0
2015	January	20.7	35.2	7.5	1
	February	20.6	34.4	6.7	0
	December	20.3	36.3	3.8	3
2016	January	21.2	39.3	8.0	6
	February	21.3	38.0	8.4	2
Total		20.8 *(m)*	37.1 *(m)*	6.2 *(m)*	31

**Table 7:** Excluded presentations during review of the medical records. The diagnosis/prognosis often had heat similar symptoms, although were discounted as not being directly associated with temperature.

Related Specialty	Total Number	Percentage	General Description
Allergy and immunology	4	9.5	Insect bites (*n* = 1); Allergic reaction (*n* = 3)
Bacterial infection/infectious disease	4	9.5	Cellulitis (*n* = 4)
Cardiovascular	2	4.8	Chest pain (*n* = 2)
Gastrointestinal	3	7.1	Constipation (*n* = 1); Gastroenteritis (*n =* 1); Epigastric pain (*n* = 1)
Musculoskeletal	18	42.9	Abdominal pain (*n* = 1); Meniscus tear (*n* = 1); General limb and joint pain (*n* = 1); Hip pain (*n* = 1); Knee pain (*n* = 3); Foot/ankle pain and swelling (*n* = 1); Wry neck (*n* = 1); Bruising (*n* = 1); Back pain (*n* = 5); Muscle sprain (*n =* 1); Arm pain (*n =* 1); Leg pain (*n* = 1)
Orthopaedic	1	2.4	Orthopaedic joint effusion (*n* = 1)
Respiratory	4	9.5	Viral illness (*n* = 2); Pneumonia (*n* = 1); Upper respiratory tract infection (*n* = 1)
Rheumatology	1	2.4	Gout (*n* = 1)
Trauma	4	9.5	Severe burn (*n* = 2); Laceration (*n* = 1); Penetrating stab wound (*n* = 1)
Urology	1	2.4	Urinary retention (*n* = 1)
Total	42	100.0	

**Table 8:** Heat related discharge diagnosis. All patients presenting with the classic signs and symptoms of heat stress and illness were treated, with the following discharge diagnosis.

Discharge Diagnosis	Patient Number	Percentage
Heat related dehydration	10	27.8
Heat related illness	9	25.0
Heat stress	7	19.4
Chest infection and heat stress	1	2.8
Heat related illness and cellulitis	1	2.8
Heat related lethargy	1	2.8
Heat related nausea and vomiting	1	2.8
Heat related syncope	1	2.8
Heat stress and dehydration	1	2.8
Heat stress related to mental disorder	1	2.8
Heat stroke and UTI	1	2.8
Heat related nausea and vomiting	1	2.8
Heat and viral illness	1	2.8
Total	36	100.0

**Table 9:** High risk criteria patient results. The old and very young were at particular risk, with strenuous outdoor activity effecting all age groups and comorbidities.

Physiological and Environmental	Total Number of Patients with the Following Risk Factors	Percentage
Strenuous outdoor physical activity during hottest day-time hours	18	33.3
Age older then >64 year old or younger then <2 year old	14	25.9
Limited access to air-conditioning	10	18.5
Medication and substance use (alcohol)	3	5.5
Mental illness	3	5.5
Physical disability/impaired mobility	3	5.5
Cognitive impairment	2	3.7
Heart and lung disease	1	1.8
Total	54	100.0

**Table 10:** Primary and secondary heat related symptoms. The strongest indicator appeared to be syncope, with a secondary symptom of nausea and/or vomiting.

Primary Heat Related Symptoms	Number	Percentage	Secondary Symptoms	Number	Percentage
Confusion	3	8.3	Confusion	2	5.6
Dizziness	3	8.3	Diarrhoea	1	2.8
Headache	3	8.3	Dizziness	9	25.0
Irritability	2	5.6	Hallucination	1	2.8
Nausea/vomiting	7	19.4	Headache	2	5.6
Syncope	17	47.2	Loss of coordination	5	13.9
Weakness	1	2.8	Nausea/vomiting	10	27.8
			Weakness	6	16.7
Total	36	100		36	100
